# *Dirofilaria repens* Testicular Infection in Child, Italy

**DOI:** 10.3201/eid2812.220424

**Published:** 2022-12

**Authors:** Sara Ugolini, Mario Lima, Michela Maffi, Francesco Pierangeli, Marzia Vastano, Tommaso Gargano, Stefania Varani, Andrea Gustinelli, Monica Caffara, Maria L. Fioravanti

**Affiliations:** Wythenshawe Hospital, Manchester, UK (S. Ugolini);; University of Manchester NHS Foundation Trust, Manchester (S. Ugolini);; IRCCS Azienda Ospedaliero–Universitaria di Bologna, Bologna, Italy (M. Lima, M. Maffi, M. Vastano, T. Gargano, S. Varani);; Ospedali Riuniti di Ancona, Ancona, Italy (F. Pierangeli);; Alma Mater Studiorum–University of Bologna, Bologna (M. Lima, T. Gargano, S. Varani, A. Gustinelli, M. Caffara, M.L. Fioravanti)

**Keywords:** dirofilariasis, parasites, vector-borne infections, zoonoses, *Dirofilaria repens*, testicular diseases, genitalia, male child, differential diagnosis, testicular neoplasms, orchiectomy, Italy

## Abstract

Testicular *Dirofilaria repens* infection was identified and confirmed by sequence analysis in a child in northeastern Italy. Because human dirofilariasis is emerging in southern and eastern Europe, this parasitic infection should be considered in the differential diagnosis of scrotal swelling in disease-endemic countries to avoid unnecessary interventions, such as orchiectomy.

Dirofilariasis is a zoonotic nematode infection that typically affects canines and other carnivores and can be transmitted to humans by Culicidae mosquitos. Dirofilariasis incidence has increased worldwide; new cases have been reported in previously nonendemic regions (*1*,*2*). This changing trend is likely related to global warming and subsequent increases in vector density and activity during the year. Canine dirofilariasis is endemic in Mediterranean countries of Europe and has 2 main etiologic agents: *Dirofilaria repens*, the main agent of subcutaneous infections, and *D. immitis*, the agent largely responsible for cardiopulmonary infections (*1*–*3*). Humans are usually dead-end hosts, and infection is mainly caused by a single immature worm (*2*). A clinical manifestation of human dirofilarial infection is pulmonary dirofilariasis, which has been predominantly detected within the Americas, although recent cases have been reported in Europe. In addition, *D. repens* nematodes cause human subcutaneous dirofilariasis (HSD), which is typical of the Old World (*1*–*3*); subcutaneous or ocular infection and infections in male genitalia, female mammary glands, lungs, liver, and mesentery have been described.

We report a case of dirofilariasis that occurred in September 2017 in a boy 13 years of age living in Bologna (northeastern Italy) who was born in Taormina (Sicily, Italy). The patient had a 5-month history of swelling in the left testicle. During initial assessment, the left testicle had a tender nodule upon palpation without associated scrotal hyperemia or inguinal lymphadenopathy. Ultrasonography showed a 1-cm, well-defined cyst containing a coiled structure with parallel echogenic walls and movement within the cyst (Figure, panel A). Subsequent magnetic resonance imaging showed the cyst was located on the testis without signs of infiltration and contained fluid mixed with tubular structures and moving artifacts (Figure, panel B). The patient was scheduled for surgical excision and histologic diagnosis. Routine preoperative laboratory tests showed normal blood cell counts: erythrocytes, 5.07 × 10^12^ cells/L; leukocytes, 5.30 × 10^9^ cells/L; and eosinophils, 0.10 × 10^9^ cells/L. Intraoperative exploration revealed a well-circumscribed, encapsulated tense nodule in the left side of the scrotum (Figure, panel C). To collect samples for histology and microbiology, we opened the cyst and found a coiled, thread-like roundworm (Figure, panel D). Further macroscopic examination indicated the worm was potentially a dirofilarial nematode. Because both ends of the worm were not visible, we identified the worm by microscopic observations and molecular sequence analysis of the remaining portions after fixing in 70% ethanol. The parasite was 423–588 µm wide with a cuticular layer 13–15 µm thick; the external surface was characterized by longitudinal ridges spaced 6–9 µm apart (Figure, panel E). We identified the nematode as a female *D. repens* by the longitudinal ridges, which we confirmed by molecular identification (*4*). We performed phylogenetic analysis of the 12S rRNA and cytochrome c oxidase subunit 1 mitochondrial genes; our specimen clustered with and was identical to *D. repens* sequences obtained from humans and dogs in Italy (Appendix Figure). The patient had an uneventful postoperative course, and no further therapy was administered. At 20-month follow-up, the patient had no residual symptoms, and ultrasonography showed no testicular abnormalities.

**Figure:**
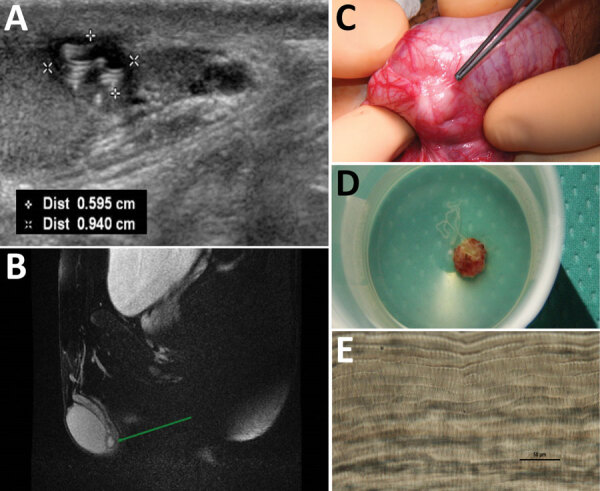
Diagnostic evaluation of *Dirofilaria repens* testicular infection in a child from Italy, a boy 13 years of age who had a 5-month history of swelling in the left testicle. A) Ultrasound scan showed a 0.5 × 0.9 cm hypoechoic cyst with moving artifacts and thread-like hyperechoic structures. B) Magnetic resonance imaging showed the cyst was located on the testis without signs of infiltration and contained fluid mixed with tubular structures and moving artifacts. C) Exploration of the scrotum before cyst excision showed a well-circumscribed, encapsulated tense nodule on the left side. D) The cyst was excised and a coiled roundworm was found in the opened capsule. E) We identified the nematode as a female *D. repens* nematode by microscopically observing typical longitudinal ridges on the body surface. Scale bar indicates 50 μm. Dist, distance.

HSD localization in male genitalia (testis, scrotum, verga, spermatic cord, and epididymis) has been previously described (*5*–*9*) and might be related to a *D. repens* tropism in response to sex hormones (*1*). Our case highlights that testicular dirofilariasis might mimic a testicular tumor and lead to unnecessary orchiectomy because of misdiagnosis. A helminthic infection should be considered in this differential diagnosis for gradual-onset testicular swelling with or without signs of inflammation, especially in endemic areas. Serologic tests for helminthic infections are performed only in specialized laboratories and are not routinely available. In addition, the accuracy and usefulness of those tests have been debated (*10*). Ultrasonography and magnetic resonance imaging can help identify features of dirofilariasis, such as dirofilarial nodules with suspicious inner hypoechoic/T1-hypointense findings, or might demonstrate moving worms (*10*). Imaging results should be consistent with a thick-walled lesion, semiliquid content with a central signal caused by the worm, and a macroscopic thread-like structure. The definitive diagnosis of HSD can only be achieved by postoperative identification of the worm by using morphologic, histologic, or molecular analysis. When malignancy cannot be excluded, an excisional biopsy is indicated for histologic diagnosis. The complete extraction of the worm is usually curative, and no specific antihelminth therapy is indicated in the absence of secondary lesions (*8*,*9*).

In conclusion, diagnoses of human dirofilariasis have increased in countries in Europe, and clinical awareness of this parasitic infection should be strengthened through education and interdisciplinary collaboration among clinicians, surgeons, and parasitologists. Clinicians should consider HSD in the differential diagnosis of subcutaneous or superficial tissue nodules of the testicles. Excisional biopsies should be performed for parasitologic, molecular, and histologic analyses to avoid invasive surgical procedures that might cause permanent reduction in quality of life, such as orchiectomy.

Appendix Additional information for *Dirofilaria repens* testicular infection in child, Italy.
