# Supplementary material for: Dirofilaria repens Testicular Infection in Child, Italy
Source: Emerg Infect Dis. 2022 Dec;28(12):2569–72. doi: 10.3201/eid2812.220424 (PMC9707574; doi:10.3201/eid2812.220424)
Supplement: Appendix — Additional information for Dirofilaria repens testicular infection in child, Italy. [file 22-0424-Techapp-s1.pdf]

Article DOI: https://doi.org/10.3201/eid2812.220424

Dirofilaria repens Testicular Infection in Child, Italy

Appendix

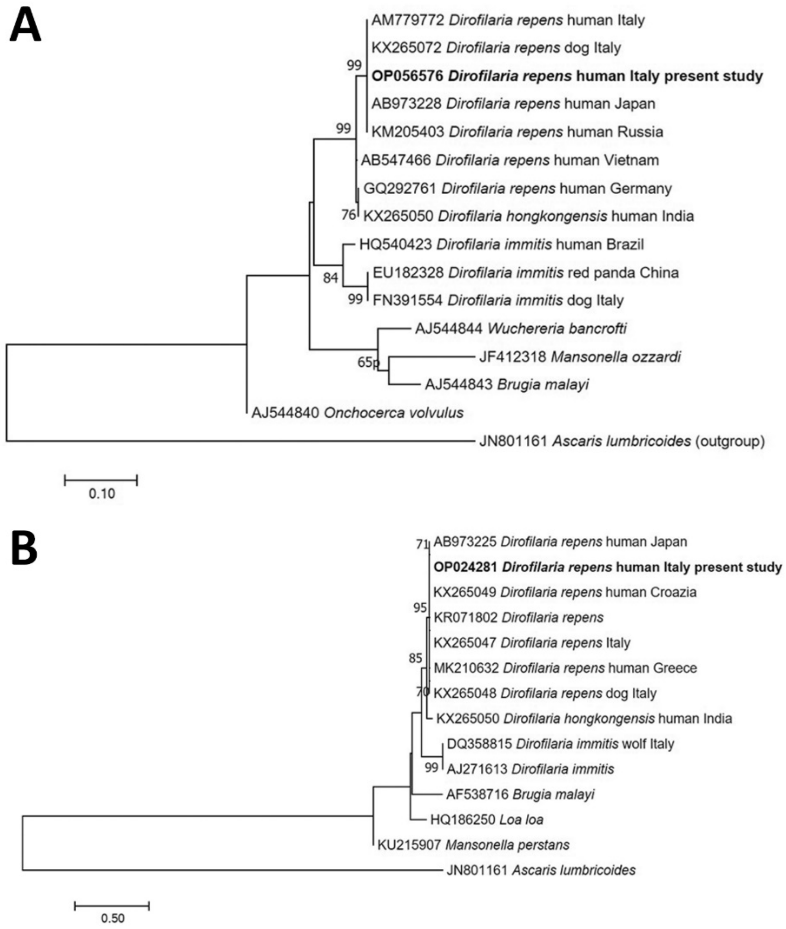

Appendix Figure. Phylogenetic analysis of the nematode *Dirofilaria repens* causing a testicular infection in a child in Italy. Phylogenetic trees were constructed by using the maximum-likelihood method. A) The sequence of the 12S rRNA mitochondrial gene from *D. repens* found in this case was compared with *D. repens* sequences from GenBank. Evolutionary history was inferred according to the Hasegawa, Kishino, Yano (HKY) model. B) The sequence of the cytochrome c oxidase subunit 1 mitochondrial gene from *D. repens* found in this case was compared with *D. repens* sequences from GenBank. Evolutionary history was inferred according to the Tamura-Nei model. Our specimen clustered with and was identical to *D. repens* sequences obtained from humans and dogs in Italy. Bootstrap values are shown at different nodes. The trees are drawn to scale; scale bars indicate nucleotide substitutions per site.
